# Plasmonic-cavity model for radiating nano-rod antennas

**DOI:** 10.1038/srep03825

**Published:** 2014-01-23

**Authors:** Liang Peng, N. Asger Mortensen

**Affiliations:** 1 Department of Electronic Engineering and Information Science, Hangzhou Dianzi University, Hangzhou, P. R. China; 2 Department of Photonics Engineering, Technical University of Denmark, DK-2800 Kongens Lyngby, Denmark; 3 Center for Nanostructured Graphene (CNG), Technical University of Denmark, DK-2800 Kongens Lyngby, Denmark

## Abstract

In this paper, we propose the analytical solution of nano-rod antennas utilizing a cylindrical harmonics expansion. By treating the metallic nano-rods as plasmonic cavities, we derive closed-form expressions for both the internal and the radiated fields, as well as the resonant condition and the radiation efficiency. With our theoretical model, we show that besides the plasmonic resonances, efficient radiation takes advantage of (a) rendering a large value of the rods' radius and (b) a central-fed profile, through which the radiation efficiency can reach up to 70% and even higher in a wide frequency band. Our theoretical expressions and conclusions are general and pave the way for engineering and further optimization of optical antenna systems and their radiation patterns.

The optical nano-antenna is a nano-sized device that can effectively radiate optical waves. Naturally, the concept also works in reverse, i.e. as a receiver of optical fields. The feasibility of nano-antennas is fostered by the advancement of nano-fabrication techniques and the concept has been attracting significant attention in recent years^1^,^2^,^3^,^4^,^5^,^6^,^7^. Based on the resonance of the localized modes, nano-antennas can strongly interact with photon fields, and hence is potentially used in quantum photonics to control the emission and absorption characteristics of single-photon devices^1^,^5^,^8^. Usually, the optical nano-antennas are designed by consulting knowhow of radio-frequency (RF) antennas which could possess the similar functionalities^1^,^7^. With such a methodology, various nanoantennas have been proposed and discussed, e.g. nano-rods, patches, bow-ties, cross-shape antennas, and Yagi–Uda type antennas^1^,^5^,^7^,^9^,^10^. For more details, we refer to Ref. 11 and references therein.

The rod-type nano-antenna (RTNA) is the geometrically simplest one. The RTNA can be matched and tuned by the combination of nano-circuits^12^,^13^,^14^, and could also be put into an array to form a specified radiation pattern, like its common radio-frequency (RF) counterparts. However, due to the lack of good conductors to support the conductive current in the optical range, nano-antennas have to utilize dispersive plasmonic building blocks. The plasmonic modes of metallic structures support strongly enhanced localized fields, which in turn stimulate the effective radiation^7^. Consequently, the nano-antennas have their peculiar characteristics, which may be drastically different from the traditional RF ones. For instance, the resonant length of dipole-like nano-rod antennas may be considerably smaller than half the free-space wavelength^15^, i. e. the typical size of the classical RF dipoles^16^. Furthermore, the reduction of radiation resistance unfortunately makes the antenna efficiency be degraded^7^,^17^.

Nano-antenna design is largely resting on radio-frequency design experience and computer-based brut-force electromagnetic simulation, while there is still a call for rigorous analytical treatments. In the following, the theoretical formulation for the radiating RTNA is derived by applying the cylindrical harmonics expansion. We derive the expressions for both the internal and the radiated fields of the RTNAs. Furthermore, we show the analytical form of both the resonance condition and the radiation efficiency of RTNAs, by drawing on their resemblance of plasmonic cavities. We emphasize that high performance requires both a plasmonic resonance and an efficient radiation of the optical energy. According to our theoretical study, high radiation efficiency, i.e. up to 70% and even higher, can be promoted by rendering a large value of the rods' radius. Finally, we discuss feeding strategies to the wide-band radiation, providing opportunities for radiation-pattern control beyond the common RF antenna design.

## Results

**Figure 1:**
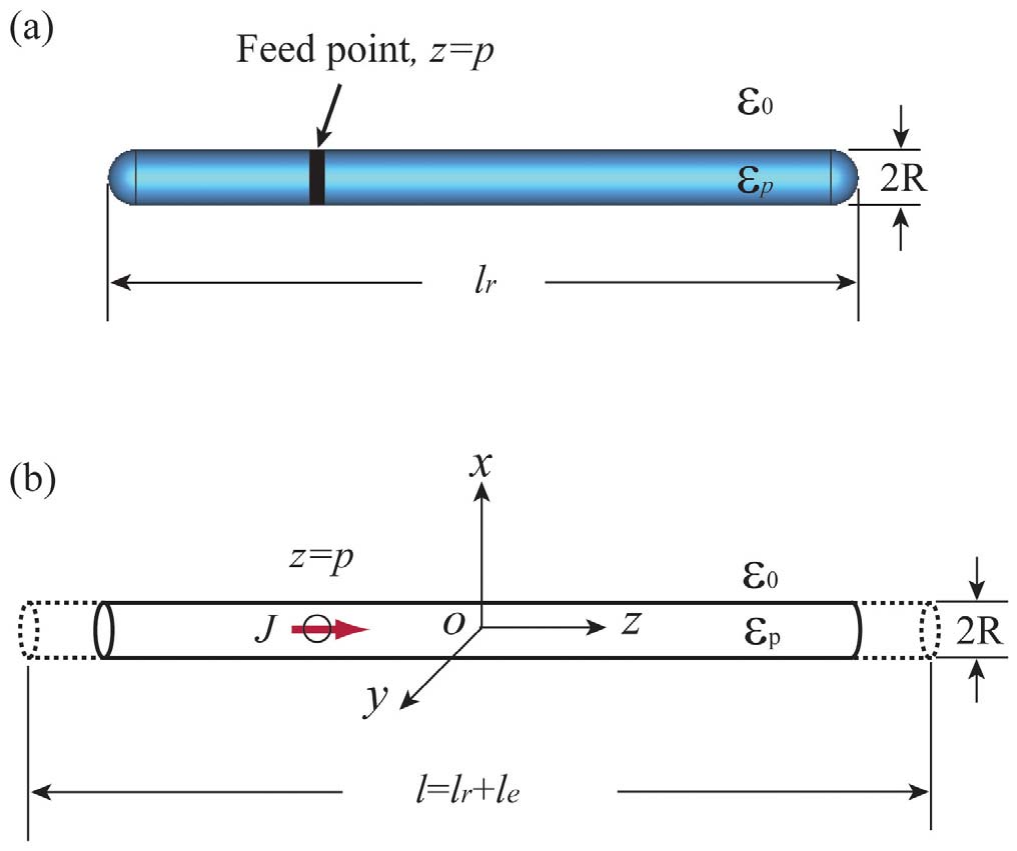
(a) The structure of a typical rod-type nano-antenna, which is supposed to be fed by a QE at *z* = *p*; the nano-antenna has a length *l_r_* with two semi-spheres at the two end-terminals. (b) The effective cylindrical cavity model of the nano-antenna; the length of the cavity is *l* = *l_r_* + *l_e_*. In both of the two sub-figures, the nano-rod has radius *R* and dielectric constant 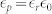.

Generally, the rod-type antenna with length (*l_r_*) is in practise comprized by a finite-length cylinder capped by two hemispheres at the terminals, as shown in Fig. 1(a). The geometrical details of the termination depend on the fabrication and nanoprocessing of the device. While geometrical features can be difficult to access in detail, cathodoluminescence experiments may implicitly provide information from reflection phases^18^. Since the cylinder has a radius (*R*) commonly being much shorter than its length (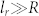), our theory will implicitly neglect the influence from the two rounded ends. Instead, we model the antenna as a finite cylinder with an effective length (*l*), see Fig. 1(b). Reflection phases from the ends of the rod will be included through an effective length^15^, i.e. *l* = *l_r_* + *l_e_*.

As a possible exciting source to the nano-antenna, a quantum emitter (QE) is placed at 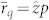 where −*l*/2 < *p* < *l*/2, see Fig. 1(a). From Ref. 8, the quantum emitter can be equivalently treated as an R-L-C series circuit. The modeling of the QE is not our main purpose, so for simplicity we assume the QE is a point current which runs in the 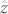 direction, i.e. ***J*** in Fig. 1(b).

Since the current ***J*** occupies a volume rather small, all of the fields inside the nano-rod can safely be considered finite on the scale of the QE. Following Ref. 19, we expand the electromagnetic fields in a basis of vector cylindrical waves. The theoretical formulation for the internal and the external fields of the RTNA is based on a plasmonic cavity model. Inside the cavity, we reasonably assume that internal fields are standing-like waves, as also supported by experimental near-field images utilizing the sub-wavelength resolution of electron-energy loss microscopy^20^. The waves inside the rod are of the form 
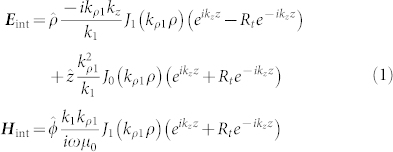
for *p* < *z* < *l*/2, and 
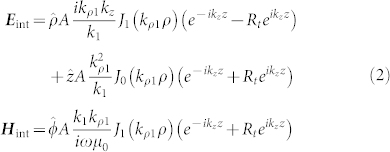
for −*l*/2 < *z* < *p*. In Eqs. (1) and (2), 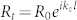 with 
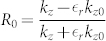
 being the reflection coefficient of the TM waves at the ends of the nano-rod. The parameter *A* ensures the continuation of the fields at *z* = *p*. In this context, the point source ***J*** is oriented in the 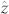 direction, and as a result 
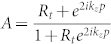
 to make *E_z_* be continuous at *z* = *p*. We note that even though Eqs. (1) and (2) define the eigenstates of the antenna, in simulation the system has to be driven by a source at *z* = *p*, in order to explore and further optimize the radiation efficiency.

### Resonance of the Nano-rod antennas

**Figure 2:**
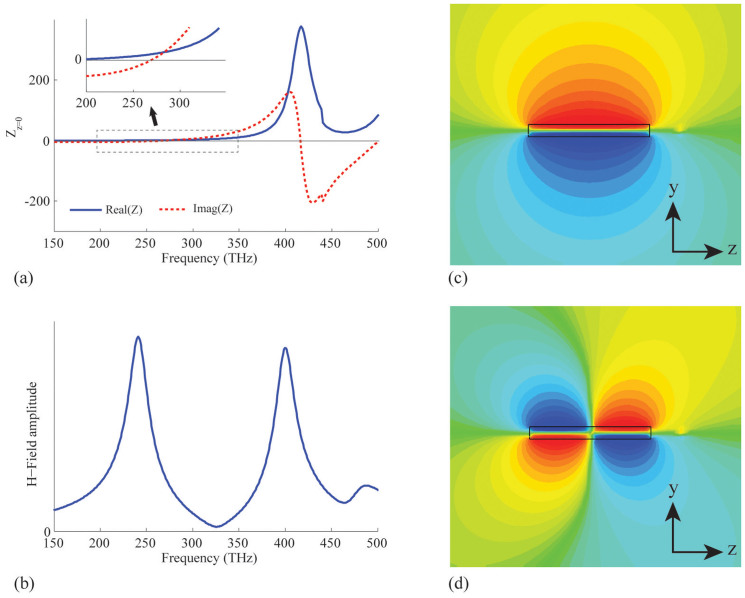
(a) *Z_z_*_ = 0_ vs. frequency. In the evaluation, *R* = 10 nm, *l* = 200 nm; (b) the simulated H-field intensity (*H_ϕ_*) at 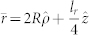; (c–d) the simulated H-field distribution of the lowest two resonant modes. In the simulation of (b–d), the exciting source is placed at *z* = *l_r_*/2 + *R*.

Although the coupling between the current and the electromagnetic (EM) modes inside the nano-antenna changes as *p* varies, efficient radiation may only occur when the plasmonic resonance makes the localized fields be enhanced. Applying the transverse resonance condition, we have 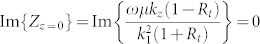, equivalent to 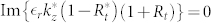. It is challenging to find the analytical solution to this problem. Numerically, we find *Z_z_*_ = 0_ for a nano-antenna made of gold with *R* = 10 nm and *l* = 200 nm, see Fig. 2. In the calculation and simulation, we use the dielectric constant for gold from Ref. 21. The nano-antenna supports two resonant modes in the observing frequency range, Fig. 2(a). Fig. 2(b) exhibits the simulated spectral response of the antenna when we place an H-field detecting probe at *ρ* = 2*R* and *z* = *l_r_*/4. The magnetic field distributions of these two modes are also shown in Fig. 2(c) and Fig. 2(d). It is seen that the second resonance has the anti-symmetric profile characteristic for dark modes, i.e. it will couple less EM power to the far field than the first one. If Im{*Z_z_*_ = 0_} = 0 is not naturally satisfied by the nano-antenna itself, the nano-circuit method can be applied to cancel the non-zero imaginary part of *Z_z_*_ = 0_, e. g. the tuning of the resonance by nano-circuit loading^14^,^17^.

### Radiation efficiency

Basically, the nano-antennas always radiate energy, but radiation is further promoted by the presence of plasmonic resonances. The radiation efficiency of the nano-antenna is not only influenced by its resonance modes but also by its practical size, which we will show below. Starting from Eqs. (1) and (2), we derive the closed-form expressions for both the radiated fields and the radiation efficiency, see the Method section.

**Figure 3:**
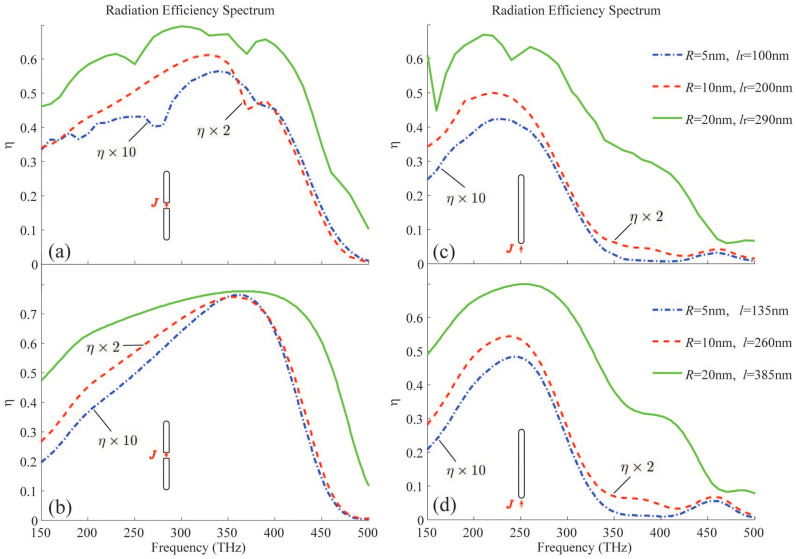
Central-fed case: the radiation efficiency from (a) simulation and (b) theoretical evaluation. End-fed case: the radiation efficiency from (c) simulation and (d) theoretical evaluation. In each of the above sub-figures, the curves for *R* = 10 nm and *R* = 5 nm have been magnified by a factor of 2 and 10, respectively.

We simulate the radiation of three RTNAs with *R* = 5 nm, 10 nm and 20 nm, which are designed to possess the similar fundamental mode at the same resonant frequency, i.e. *f_n_*_ = 0_ ≈ 260 THz. We first assume that the nano-rod antenna is fed at the center of the antenna (*p* = 0). The radiation efficiency spectra extracted from the simulated results are shown in Fig. 3(a). We see that the three antennas have remarkably different efficiencies, i.e. the larger the radius the higher the efficiency. This is because the RTNA with 20 nm radius has a resonant length longer than the two others, which enhances the radiation power and the effective radiation resistance^7^,^17^. The radiation efficiency evaluated from our theoretical formulas are shown in Fig. 3(b). In the calculation, *l* is the effective length being longer than the practical length *l_r_*. Next, we assume the nano-rod antenna is fed by ***J*** located in the vicinity of one of the terminals of the nano-rod. In our simulation, the current source is placed just outside the rod structure, i.e. *p* = *l_r_*/2 + *R* or *p* = −*l_r_*/2 − *R*. However, in our theoretical evaluation, *p* = *l*/2 or *p* = −*l*/2 is assumed. The simulated efficiency for the above three nano-rod antennas are shown in Fig. 3(c), while the theoretical estimated curves are shown in Fig. 3(d). We see that the efficiency can reach up to 70% and even higher if we further enlarge the radius *R*.

**Figure 4:**
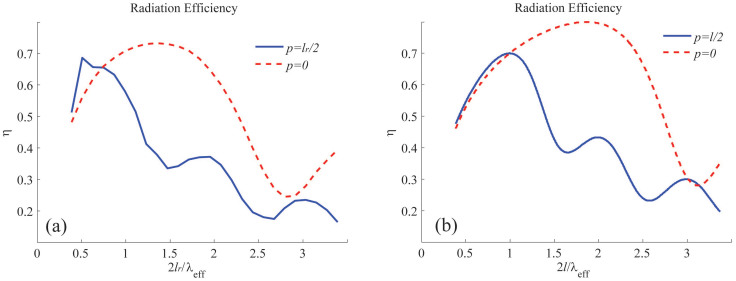
(a) The simulated and (b) the theoretically estimated radiation efficiency of rod-type nano-antenna with varying the antenna length. The solid line is the end-fed case, the dashed line is the central-fed case. For both the two cases, *R* = 20 nm.

**Figure 5:**
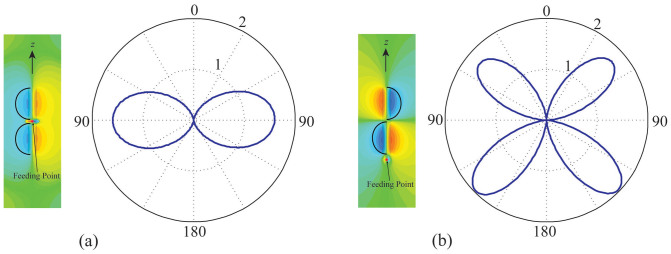
The magnetic field (*H_ϕ_*) distribution and the radiation directivity (in E-plane) of a rod-type nano-antenna with *R* = 20 nm, *l_r_* = 290 nm. (a) The central-fed case; (b) the end-fed case. The operating frequency is 400 THz, equivalently *l_r_* ≈ *λ*_eff_.

We also see from Fig. 3 that the radiation efficiency may be improved if we increase the length of the RTNA. To show this in more detail, we keep the radius of the rod to be 20 nm, but vary the length. Fig. 4(a) shows the simulated efficiency, while Fig. 4(b) shows that of our theoretical estimation. It is obvious that for the central-fed case, the efficiency may be optimized by moderately increasing the *l_r_* (or *l*). This is because the current ***J*** implies that the field distribution has to be symmetric in the two balanced branches. Technologically, this increases the resonant length and consequently promotes the radiation resistance. However, for the end-fed case without any benefit from the feeding profile, the unbalanced structure makes the fast oscillation of *η* as *l_r_* increases. In Fig. 5, the magnetic near-field distribution and the radiation directivity of a RTNA with *R* = 20 nm, *l_r_* = 290 nm, and *f* = 400 THz (i.e. *l_r_* ≈ *λ*_eff_) are simulated. It is seen that the radiation pattern by the magnetic symmetric mode in Fig. 5(a) is much different from the anti-symmetric one in Fig. 5(b). Again, we emphasize that a central-fed antenna can radiate more efficiently than an end-fed one beyond the antenna's self-resonant frequency, see Fig. 3. It should be noticed that the RTNA alone may not resonate at *f* = 400 THz, however the nano-circuits can be applied to make the RTNAs tuned^8^,^13^. Moreover, the wide-band radiation may benefit from the central-feeding technique, i.e. the high efficiency band in Fig. 3(a) and Fig. 3(b) is much wider than that shown in Fig. 3(c) and Fig. 3(d).

## Discussion

In Fig. 3, we show the spectral dispersion of the antennas' efficiency. It is evident that the efficiency of the antenna is indeed influenced by the plasmonic modes. The reason is that the optical power is hardly coupled to the free space beyond the resonance mode, but the Ohmic loss is always present (see *P*_rad_ and *P*_Ohm_ in the Method part). Meanwhile, being influenced by the singularity of the source, the dissipation of power is enhanced by the existence of the evanescent modes, hence the simulations exhibit lower efficiency than in the theoretical estimation. In addition, we may notice that the typical radius of the RTNA is comparable to the skin-depth of the metal, if we want the antenna efficiency to be 70% or even higher. In such a case, the resonant length of the nano-rod may reach half the free-space wavelength.

Considering Fig. 5, the feeding occurs in the vicinity of the nodal points in the field patterns. In an RF context, this would be an unfruitful feeding strategy. However, in the optical range such feeding is feasible and allows radiation-pattern control beyond what is possible in the RF regime.

In conclusion, through a cavity model, we have derived the analytical expressions for both the internal and the radiated fields, as well as the radiation efficiency of RTNAs. With the insight provided by our theoretical formulation and simulations, we show that radiation of the nano-rod antennas may take advantage of a length comparable to the wavelength, which indicates that the radius of the nano-rod can not be too small compared to the skin-depth of the metal. Hence, some of the existing nano-antennas with sharp terminals like the bow-ties, may have profiles advantageous for field enhancement, while electromagnetic energy is radiated inefficiently.

We also show that the antenna's bandwidth may benefit from a central-fed profile, by which wide-band radiation may be realized with properly matching of the antenna by nano-circuits. Finally, the feeding technique can be used for radiation pattern control, with possible applications in the emerging field of quantum plasmonics^22^.

## Methods

### Harmonics expansion of the local fields

From Ref. 19, the electromagnetic waves inside and in the surroundings of the infinitely long rod can be represented into a superposition of vector cylindrical waves, 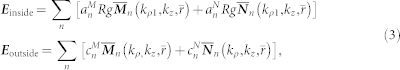
with 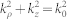, 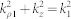, 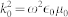, 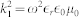. Here, the *Regular* wave functions are given by 
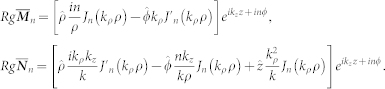


For 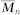 and 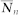, the Bessel function is to be replaced by the Hankel function of the first kind. In Eq. (3), 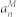, 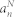, 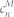 and 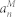 are unknown coefficients that can be determined by satisfying the boundary conditions.

The point source ***J*** is placed at 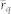 which satisfies 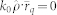. Hence the lowest radiation mode of the nano-rod antenna has a rotationally symmetric profile, so that we can focus on the resonance mode with *n* = 0. If we apply the boundary conditions to the surface of the nano-rod, i.e. let *ρ* = *R*, we get 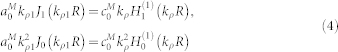
for TE modes, and 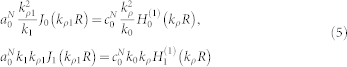
for TM modes. The non-trivial solution implies that 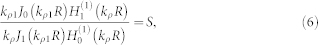
with *S* = 1 for TE modes and 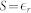 for TM modes, respectively. Generally, the magnetization in the 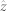 direction makes 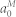 and 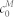 non-zero. However, for the RTNA considered here, 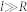 allows us to just consider the electric polarization in the low frequency range, which means that only 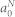 and 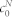 can be finite. With 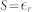, Eq. (6) can be solved numerically or semi-analytically. In particular, the shortening of the effective wavelength in the *z*-direction is predicted by the increasing of *k_z_*^15^.

### Resonance condition

According to the conventional EM engineering, if we treat the nano-rod as a finite transmission line in the *z*-direction, a transverse resonance occurs when the complex conjugate matching condition is satisfied. Consequently, the impedance (Z) at *z* = 0 should be purely resistive, with consideration to the symmetry of the RTNAs. Hence we naturally have 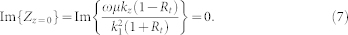


### The radiation efficiency

The radiated fields associated with the polarization can be found in Ref. 16, which we here rewrite as 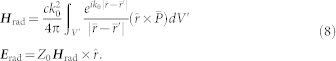
Here, 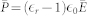 is the polarization at 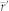, while *V*′ is the volume occupied by the nano-rod. In the far-field zone, 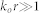, we have 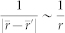 and 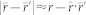. Still, the radius of the nano-rod is small enough, so that 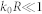 holds. Substituting Eqs. (1) and (2) into Eq. (8), and taking the commonly employed far-field approximation, we get 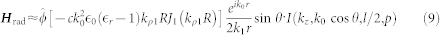
with 
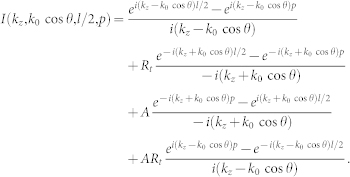


To find the efficiency of the nano-antenna, we define 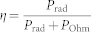, with *P*_rad_ being the total power radiated and *P*_Ohm_ being the power dissipated on the nano-rod structure due to the inherent Ohmic resistance. Applying the closed surface integral of the Poynting power flow, i.e. 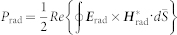, we get 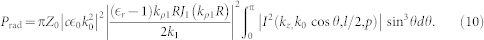
Again, from the classical EM theory, the loss on the nano-rod can be evaluated by 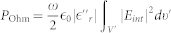, which in detail reads 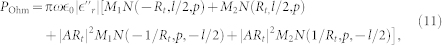
with 
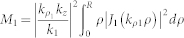
, 
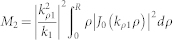
, and 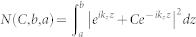. The latter evaluates to 
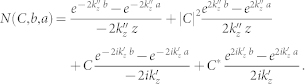


In the above two expressions, 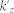 and 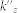 are the real and the imaginary parts of *k_z_*, respectively. The expressions for both *P*_rad_ and *P*_Ohm_ are too complex to accomplish further simplification, so in the above context we have to evaluate the antenna efficiency by numerical means.

## Author Contributions

L.P. conceived the idea and performed the analytical and numerical calculations. L.P. and N.A.M. participated in the analysis of the results and in the subsequent writing of the manuscript.
